# The Impact of Snapchat Use on Self-Image and Inclination Toward Cosmetic Procedures in Saudi Arabia

**DOI:** 10.7759/cureus.32456

**Published:** 2022-12-12

**Authors:** Basem K Alhusaini, Futoun Z Sharaf, Reem O Alqthmi, Nouf T Alotaibi, Maysan B Ballaji, Huda M Mohammed

**Affiliations:** 1 Department of Plastic and Reconstructive Surgery, King Fahad General Hospital, Al Madinah, SAU; 2 College of Medicine, Al-Rayan Colleges, Madinah, SAU; 3 College of Medicine, Umm Al-Qura University, Makkah, SAU; 4 College of Medicine, Alzaiem Alazhari University, Khartoum, SDN

**Keywords:** body image, acss, social media, snapchat, cosmetic surgery

## Abstract

Background

Exposure to social media gives individuals more significant body image concerns, and many studies have emphasized that people with a negative body image are more interested in cosmetic surgery. So, increased exposure to celebrities with aesthetic images and exposure to cosmetic Surgery information and advertisements lead to negative body image in the exposed populations.

Aim

This study aimed to determine the impact of social media on self-image inclination toward cosmetic procedures.

Subjects and methods

This is a cross-sectional study conducted among an adult population in the Kingdom of Saudi Arabia. A self-administered questionnaire was distributed among participants using social media platforms. the questionnaire was sought information on socio-demographic characteristics (age, gender, education, etc.), the influence of social media on the decision to undergo cosmetic surgery, and the Acceptance of Cosmetic Surgery Scale (ACSS).

Results

Among the 1064 participants recruited, 41.4% were aged less than 25 with females outnumbering men substantially (82.1). Twenty-seven of the respondents increased their desire to undergo a cosmetic procedure due to advertisements or publications posted on social media. The total mean ACSS score was 3.36 (SD 1.69) out of 10 points, indicating lower acceptance. The socio-demographic variables associated with increased ACSS scores were being older, female, educated, having been married, and previous history of cosmetic surgery.

Conclusion

Despite the influence of Snapchat on body image, there was a low inclination to undergo cosmetic surgery among the general population in Saudi Arabia. The tendency to undergo cosmetic surgery tends to increase due to the influence of increased social media following, social media influencers, and advertisements and publications posted on social media platforms. More research is warranted to determine the perspectives of the general public regarding the influence of social media platforms on the inclination to undergo cosmetic surgery procedures for the enhancement of body appearance.

## Introduction

Aesthetic procedures are among the most commonly performed surgeries in the medical field. These procedures have been growing in popularity in our nation due to multiple factors including body image dissatisfaction and the pursuit of perfection, in addition to the growing influence of social media [1]. Cosmetic surgery focuses on enhancing appearance including surgical procedures such as blepharoplasty, rhinoplasty, and breast augmentation, as well as nonsurgical procedures, such as chemical peels and botulinum toxin injections. Plastic surgery by definition deals mainly with the surgical repair or restoration of an injured, lost, diseased, defective, or misshapen part or area, and typically involves tissue grafting from one part of the body to another [2]. Social media is gaining popularity worldwide as a method for advertising cosmetic treatments. It is necessary to determine the role of social media in influencing a person’s decision to undergo a cosmetic procedure [3]. The 2017 annual American Academy of Facial Plastic and Reconstructive Surgery survey found that 55% of the patients are willing to undergo a cosmetic procedure to modulate their selfie looks, which has led to a phenomenon called “Snapchat dysmorphia", where patients seek surgeries that mimic the effect of a Snapchat filter [4]. Holland et al. highlighted the relationship between spending significant time on social media platforms or engaging with more appearance-related content (e.g., images) on social media and more significant body image concerns [5]. According to [6], Furnham and Levitas, increasing media exposure, poor self-esteem and life satisfaction increase the likelihood of undergoing cosmetic surgery. A study found [7] that 65.7% of the patients who attended Saudi Arabian plastic surgery centers were motivated by cosmetic surgery outcomes before and after photographs uploaded on aesthetic surgeons' social media accounts. The most frequently used social media platform among patients who underwent rhinoplasty surgery was Snapchat (73.7%), followed by Instagram (12.7%) [8]. So, more exposure in celebrities with improved imaging and exposure to cosmetic Surgery information and advertisements lead to negative body image in the exposed populations.

## Materials and methods

This is a cross-sectional study conducted among an adult population in the Kingdom of Saudi Arabia. A self-administered questionnaire was distributed among participants using social media platforms (Snapchat, Twitter, WhatsApp, Telegram). The questionnaire was sought information on socio-demographic characteristics (age, gender, education, etc.). The participants answered 27 valid questions from a previous study about the influence of social media on the decision to undergo cosmetic surgery and the Acceptance of Cosmetic Surgery Scale (ACSS). The study duration was conducted from May 24, 2022 to July 31, 2022 after being approved by the Institutional Review Board (IRB); the General Directorate of Health Affairs in Madinah issued approval National Registration Number with NCBE-KACST, KSA: (H-03-M-84).

Inclusion and exclusion criteria

The inclusion criterion was anyone 18 years and above, living in Saudi Arabia and having social media platforms (Snapchat). People living outside Saudi Arabia or younger than 18 years old or who do not have Snapchat were excluded from the study.

Statistical analysis

Descriptive statistics were calculated and summarized as numbers, percentages, mean and standard deviation. The differences in the score of ACSS relating to the socio-demographic characteristics and the impact of social media on a decision for cosmetic surgery were ascertained through a Mann-Whitney Z-test. Normality test was performed using Shapiro Wilk test and Kolmogorov-Smirnov test. The overall acceptance score followed the non-normal distribution. Therefore, the non-parametric test was applied. A p-value of <0.05 (two-sided) was used to indicate statistical significance. All data analyses were performed using the Statistical Packages for Software Sciences (SPSS) version 26 (IBM Corporation, Armonk, NY).

## Results

Among the 1,064 participants recruited, 41.4% were aged less than 25 with females outnumbering men substantially (82.1). Twenty-seven percent of the respondents increased their desire to undergo the cosmetic procedure due to advertisements or publications posted on social media. The total mean ACSS score was 3.36 (SD 1.69) out of 10 points indicating, lower acceptance. Socio-demographic variables associated with increased ACSS scores were being older, female, educated, having been married, and previous history of cosmetic surgery.

In total, 1,064 participants were recruited. Table 1 presents the socio-demographic characteristics of the participants. The most common age group was less than 25 years (41.1%). Most participants were females (82.1%) and were university degree holders (74.9%). With respect to marital status, 49.1% were single. The previous history of cosmetic history was reported by 3.5% and the most common type of plastic surgery was cosmetic surgery (37.8%).

**Table 1:** Socio-demographic characteristics of participants (n=1,064)

Study variables	N (%)
Age group	
<25 years	437 (41.1%)
25 – 35 years	234 (22.0%)
36 – 45 years	214 (20.1%)
46 – 55 years	144 (13.5%)
>55 years	35 (03.3%)
Gender	
Male	190 (17.9%)
Female	874 (82.1%)
Educational level	
Primary school	10 (0.90%)
Intermediate school	40 (03.8%)
Secondary school	217 (20.4%)
University degree	797 (74.9%)
Marital status	
Single	522 (49.1%)
Married	502 (47.2%)
Divorced	27 (02.5%)
Widowed	13 (01.2%)
Previous history of cosmetic surgery	
Yes	37 (03.5%)
No	1027 (96.5%)
Type of plastic surgery ^(n=37)^	
Liposuction/Tightening	05 (13.5%)
Cosmetic surgery	14 (37.8%)
Facelift blepharoplasty-face contouring	04 (10.8%)
Others	14 (37.8%)

**Figure 1:**
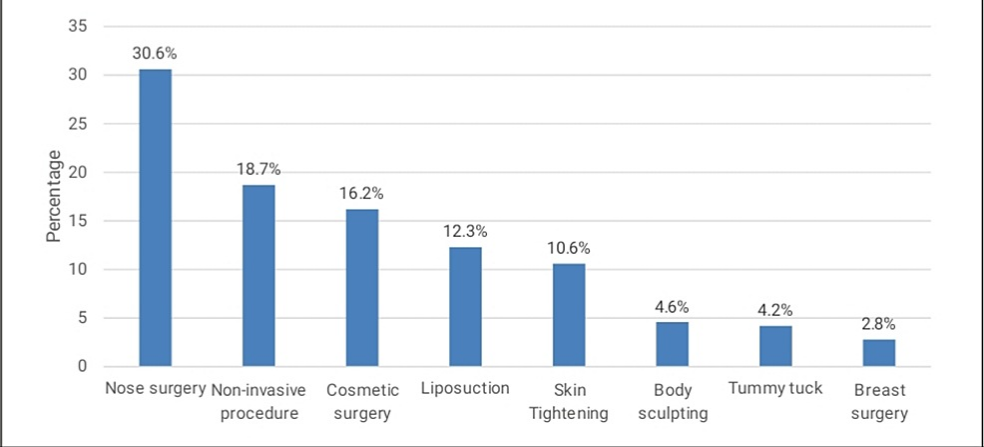
Most preferred type of surgery to be undergone in the future

In Figure 1, the most preferred type of surgery to be undergone as per the future was nose surgery (30.6%), followed by non-invasive procedures (18.7%) and cosmetic surgery (16.2%).

Regarding the influence of social media on the decision to undergo cosmetic surgery (Table 2), 37.1% expressed that the pictures on Snapchat before and after affect their desire to undergo cosmetic intervention while 44.1% indicated that undergoing cosmetic surgery is a way to increase followers on the Snap chat account. The proportion of participants who believed that undergoing a cosmetic procedure was popular among social media influencers was 79%. In addition, 27% reported being influenced to undergo cosmetic surgery by advertisements or publications that posted on social media sites.

**Table 2:** Influence of social media on the decision to undergo cosmetic surgery (n=1,064)

Statement	N (%)
Do the before and after pictures on Snapchat affect your desire for cosmetic intervention, whether surgical or otherwise?	
Yes	395 (37.1%)
No	669 (62.9%)
Is undergoing cosmetic surgery a way to increase followers on your Snapchat account?	
Yes	469 (44.1%)
No	595 (55.9%)
Is undergoing a cosmetic procedure a popular procedure among social media influencers?	
Yes	841 (79.0%)
No	223 (21.0%)
Are you persuaded to undergo a cosmetic surgery from advertisements or publications that appear on social media platforms?	
Yes	287 (27.0%)
No	777 (73.0%)

The assessment of ACSS comprises three subscales: intrapersonal subscale, social subscale and consider subscale as furnished in Table 3. Pertaining to the intrapersonal subscale, the mean rating was highest in the statement “Plastic surgery positively affects people by giving them a sense of satisfaction self-satisfaction” (mean score: 4.30) while it was the lowest in the statement “People who are not satisfied with their appearance should consider plastic surgery as an option” (mean score: 3.28). The total mean score of the intrapersonal subscale was 3.73 (SD 1.77). Regarding the social subscale, the mean score was highest in the statement “I will consider plastic surgery if it will benefit me in my career” (mean score: 3.20), while it was the lowest in the statement “It is possible for me to get a plastic surgery to look younger” (mean score: 2.87). The total mean score social subscale was 3.01 (SD 1.88).

**Table 3:** Descriptive statistics of the Acceptance of Cosmetic Surgery Scale (ACSS) (n=1,064) Response has a range from “Disagree a lot” coded as 1 to “Agree a lot” coded as 7.

ACSS statement	Mean ± SD
Intrapersonal subscale score	3.73 ± 1.77
It makes more sense to resort to plastic surgery instead of spending your life feeling bad about your appearance	3.50 ± 2.15
Plastic surgery positively affects people by giving them a sense of self-satisfaction with themselves	4.30 ± 2.04
People who are not satisfied with their appearance should consider plastic surgery as an option	3.28 ± 1.99
You should try cosmetic surgery if it will make you happier about your appearance	3.29 ± 2.13
Cosmetic surgeries can increase a person's self-confidence	4.27 ± 2.16
Social subscale score	3.01 ± 1.88
I would seriously consider getting plastic surgery if my parents think it's a good idea	3.05 ± 2.19
It is possible for me to get plastic surgery to look younger	2.87 ± 2.12
I will consider plastic surgery if it will benefit me in my career	3.20 ± 2.21
I will undergo plastic surgery if it makes me more attractive to my partner	2.97 ± 2.22
If a simple plastic surgery would make me more attractive to others, I would consider doing it	2.95 ± 2.15
Consider subscale score	3.33 ± 1.85
It is possible that I will have one of the plastic surgeries in the future	3.21 ± 2.18
I would consider getting plastic surgery if it was free	3.06 ± 2.25
I will undergo plastic surgery if I know that it is not painful and has no side effects	3.55 ± 2.39
Sometimes I think about plastic surgery	3.33 ± 2.28
I will never do any plastic surgery	4.49 ± 2.20
Overall acceptance score	3.36 ± 1.69

Finally, for the consider subscale, the mean score was the highest in the statement “I will never undergo any plastic surgery” (mean score: 4.49), while it was the lowest in the statement “I would consider getting a plastic surgery if it was free” (mean score: 3.06). The total mean score for consider subscale was 3.33 (SD 1.85). The overall mean score for ACCS was 3.36 (SD 1.69).

When measuring the differences in the score of ACSS in relation to the socio-demographic characteristics and the influence of social media on cosmetic surgery (Table 4), it was found that a higher acceptance score was more associated with the older age group (Z=3.027; p=0.002), gender female. (Z=2.117; p=0.034), university degree or higher (Z=3.057; p=0.002), having been married (Z=3.515; p<0.001), previous history of cosmetic surgery (Z=4.861; p<0.001), influence by the Snapchat pictures for cosmetic intervention (Z=13.597; p<0.001), the influence of cosmetic surgery as a way to increase followers on Snapchat account (Z=2.868; p=0.004), undergoing a cosmetic procedure due to the influence of social media influencers (Z=3.253; p=0.001), and undergoing cosmetic surgery due to the influence of advertisements or publications that appear on social media platform (Z=11.131; p<0.001).

**Table 4:** Differences in the score of overall acceptance according to the participants’ socio-demographic characteristics and the influence of social media on cosmetic surgery (n=1,064) § P-value has been calculated using Mann-Whitney Z-test. ** Significant at p<0.05 level.

Factor	Acceptance Score (7) Mean ± SD	Z-test	P-value ^§^
Age group			
≤35 years	3.22 ± 1.62	3.027	0.002 **
>35 years	3.59 ± 1.80		
Gender			
Male	3.15 ± 1.77	2.117	0.034 **
Female	3.40 ± 1.68		
Educational level			
Secondary or below	3.09 ± 1.69	3.057	0.002 **
University degree	3.45 ± 1.69		
Marital status			
Never been married	3.16 ± 1.62	3.515	<0.001 **
Been married	3.54 ± 1.75		
Previous history of cosmetic surgery			
Yes	4.74 ± 1.51	4.861	<0.001 **
No	3.31 ± 1.68		
Do the before and after pictures on Snapchat affect your desire for cosmetic intervention, whether surgical or otherwise?			
Yes	4.29 ± 1.59	13.597	<0.001 **
No	2.81 ± 1.51		
Is undergoing cosmetic surgery a way to increase followers on your Snapchat account?			
Yes	3.53 ± 1.70	2.868	0.004 **
No	3.22 ± 1.68		
Is undergoing a cosmetic procedure a popular among social media influencers?			
Yes	3.43 ± 1.66	3.253	0.001 **
No	3.08 ± 1.83		
Are you influenced to undergo cosmetic surgery by advertisements or publications that appear on social media platforms?			
Yes	4.33 ± 1.61	11.131	<0.001 **
No	2.99 ± 1.59		

## Discussion

The present study sought to determine social media's influence on self-body image inclination to undergo cosmetic procedures. The findings of this study indicate that social media influencers are considered the most dominant factor that influences our population to consider undergoing cosmetic enhancement. Approximately 80% showed great interest in undergoing cosmetic procedures because of prominent personalities on the internet prompted by the goal to achieve more social media followers (44.1%) while filtering self-image on Snapchat (37.1%), and advertisements or publications posted online (27%) were also significant contributing factors. These findings almost mirrored the study of Arab et al. [3]. According to their reports, advertisements influenced nearly half of the participants in their decision to take cosmetic procedures. Furthermore, two-thirds of the respondents were likely or certainly enticed to undergo cosmetic enhancement in the future and more than half showed interest in the blogs related to cosmetic procedures initiated by either plastic surgeons or social influencers. Interestingly, more than a quarter usually felt degraded when they compared themselves to celebrities on social media. Another study by Alghonaim et al. [1] noted that the price of cosmetic procedures is a big consideration in choosing to undergo such procedures expressed by 77% of the participants. Also, most of them believed that cosmetic specialized accounts on social media are beneficial (97%); however, 77.8% thought that such accounts did not provide reliable information about cosmetic procedures, while 68% were influenced by social media personalities. It is undeniable that the presence of social media platforms alters the perspective of users on all accounts including perceptions toward cosmetic enhancement procedures. Albeit, such procedures can enhance physical appearance, the occurrence of complications postoperatively is a big consideration, since it could change everything, and worst of all it could affect the patient’s quality of life.

The mean ACSS score was 3.36 (SD 1.69) out of 10 points suggesting a lower acceptance for cosmetic surgical procedures. Among ACSS subscales, the intrapersonal subscale has the highest mean (mean score: 3.73), followed by the consider subscale (mean score: 3.33), and the social subscale (mean score: 3.01). These results are slightly lower than that of Dujmović et al. [9]. Based on their accounts, the overall mean score of ACSS was 3.73. Regarding subscales, intrapersonal was the highest (mean score: 4.63) followed by the consider subscale (mean score: 3.66) and the social subscale was the lowest (mean score: 2.89). The ACSS questionnaire is an important indicator of participants' willingness to undergo such surgery, our results indicate a mediocre acceptance among our population which may be due to social factors, consideration factors, and intrapersonal factors.

Data in our study suggest that the increased inclination to undergo cosmetic surgery was mostly seen in older participants, females, being educated, having been married, and with a previous history of cosmetic surgery. In Croatia [9], a study documented that females were more associated with high scores on five ACSS scales, but there were no significant differences displayed in three ACSS subscales and the overall ACSS score. Also, women observed a significant inverse correlation between the ACSS subscale score and satisfaction. However, in the Eastern region [10], level of education and marital status were found to be the relevant factors of the ACSS social domain which was also consistent with our results.

Moreover, the willingness to undergo cosmetic procedures increased after filtering self-image on Snapchat, the desire to increase Snapchat followers, the influence of social media personalities, and the influence of advertisements or publications posted on social media platforms. In a study conducted in Riyadh [3], results showed that watching cosmetic surgery-related ads on social media, extended use of social media platforms, and having negative self-views when using social media are assumed to increase the interest in undergoing cosmetic enhancement in the future. Consistent with previous findings, an increase in time spent using social media influenced the desire of UK university students to undergo such procedures [11], while dissatisfaction with physical appearance and following different social media personalities were also significant factors. However, in a study by Chen et al. [4], social media engagement had a direct association with the desire to get cosmetic surgery. Tinder are Snapchat and Snapchat photo filters different? were shown to have higher total ACSS scores while increased consideration had been detected in users of VSCO and Instagram photo filters.

## Conclusions

Despite the influence of Snapchat on body image, there was a low willingness to undergo cosmetic surgery among the general population in Saudi Arabia. Further, the willingness to undergo cosmetic surgery tends to increase due to the influence of increased social media followers, social media influencers, and advertisements and publications posted on social media platforms. More research is warranted to establish the perspectives of the general public regarding the influence of social media platforms on undergoing cosmetic procedures for body appearance enhancement.
